# Supplementary figures and images for: A registration strategy to characterize DTI-observed changes in skeletal muscle architecture due to passive shortening
Source: PLoS One. 2025 Mar 10;20(3):e0302675. doi: 10.1371/journal.pone.0302675 (PMC11892864; doi:10.1371/journal.pone.0302675)

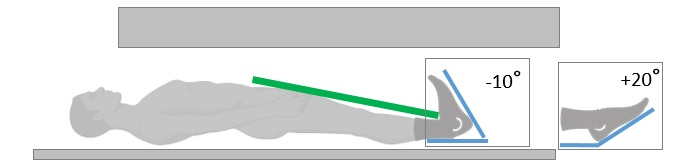

Supplement: S1 Fig — In green is shown the anterior receive coil positioned on top of the lower extremities. (TIF) [file pone.0302675.s001.tif]

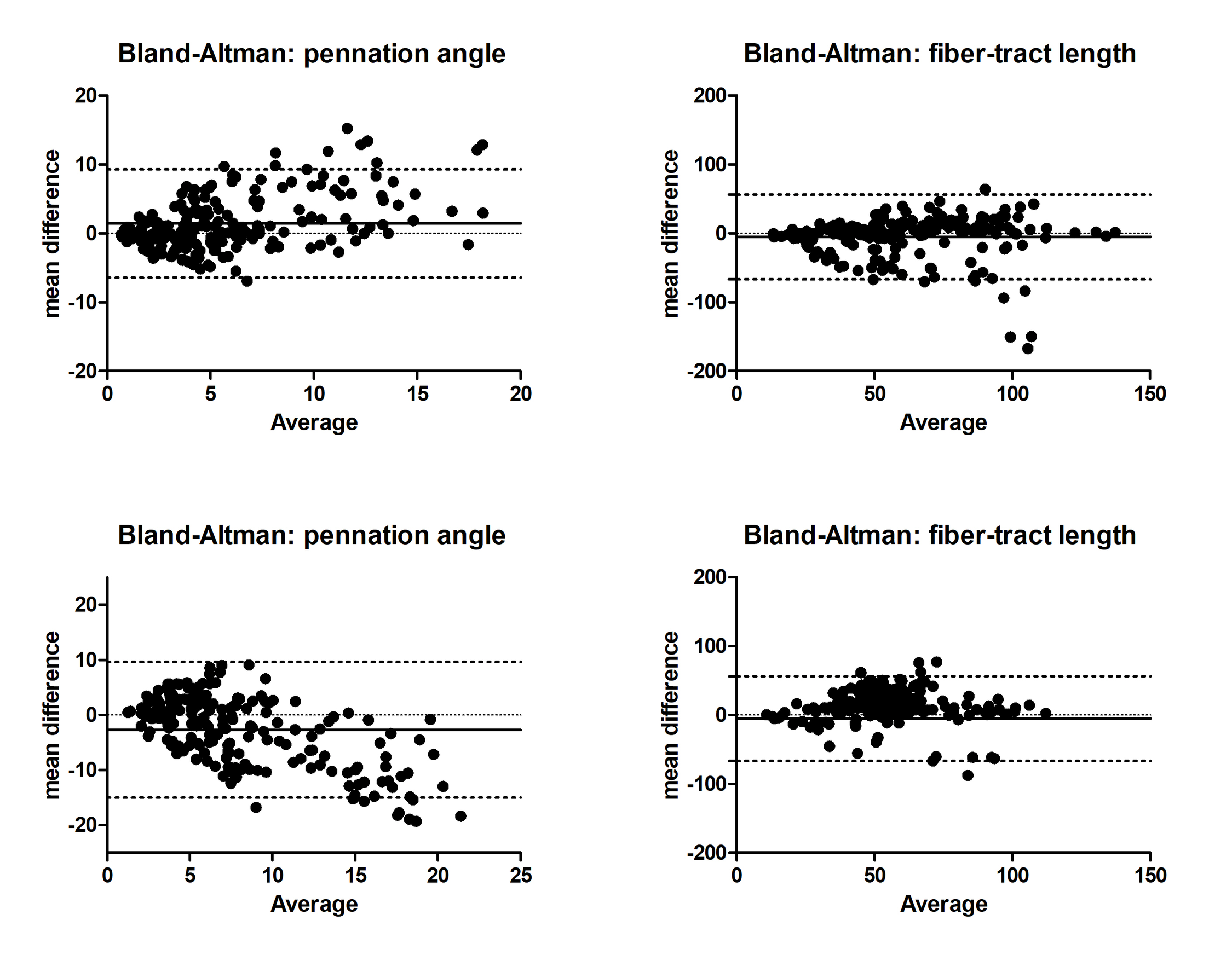

Supplement: S2 Fig — Note differences in Y-axis scales between the left and right panels. (TIF) [file pone.0302675.s002.tif]

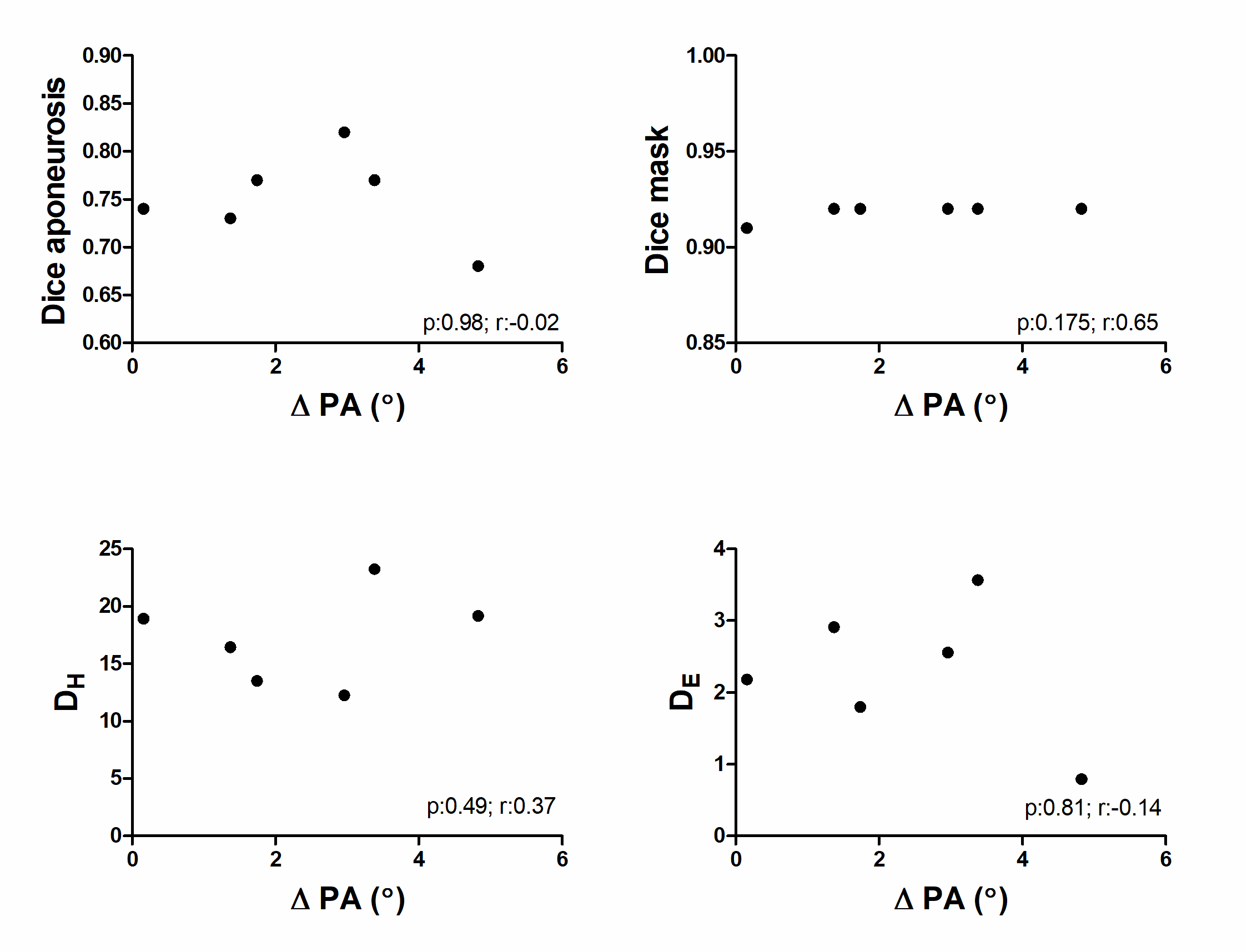

Supplement: S3 Fig — (TIF) [file pone.0302675.s003.tif]

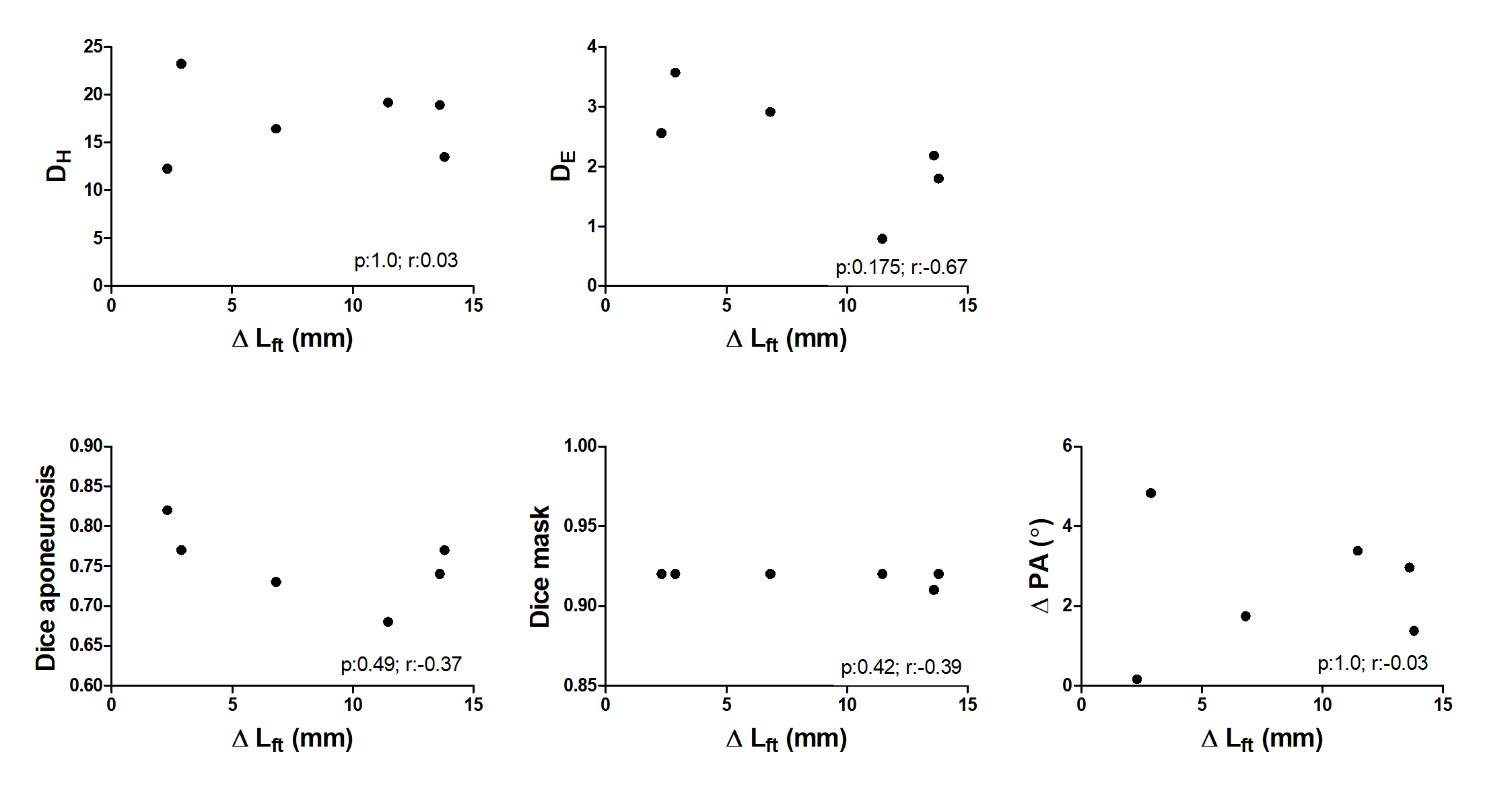

Supplement: S4 Fig — (TIF) [file pone.0302675.s004.tif]

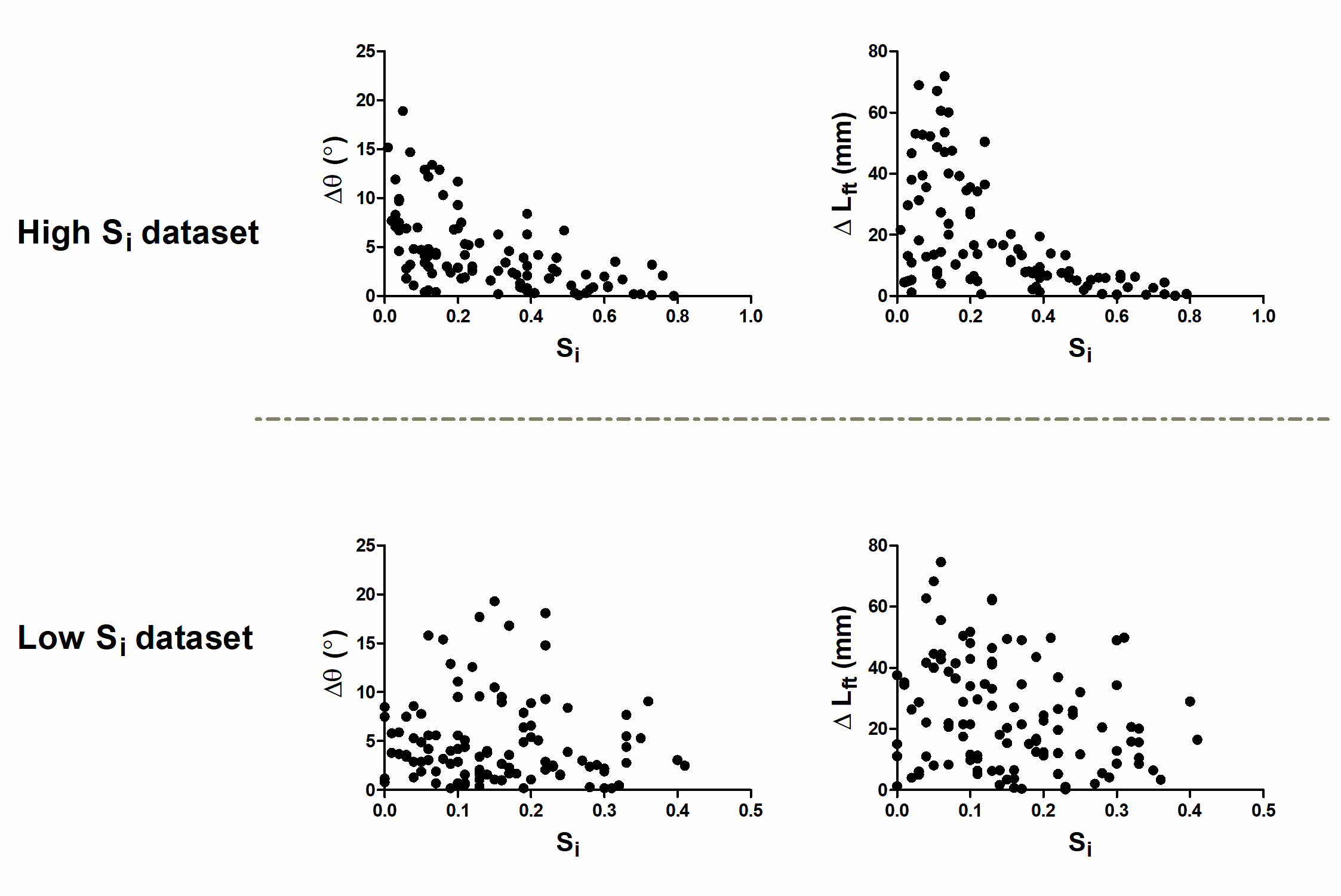

Supplement: S5 Fig — (TIF) [file pone.0302675.s005.tif]
